# Development of Teacher Rating Scale of Risky Play for 3- to 6-Year-Old Pre-Schoolers in Anji Play Kindergartens of East China

**DOI:** 10.3390/ijerph19052959

**Published:** 2022-03-03

**Authors:** Wenqi Lin, Jianfen Wu, Yunpeng Wu, Hongli He

**Affiliations:** 1 Jing Hengyi School of Education, Hangzhou Normal University, Hangzhou 311121, China; 2019111004025@stu.hznu.edu.cn; 2 School of Teacher Education, Dezhou University, Dezhou 253023, China; 3 Zhejiang Education Press Group, Hangzhou 310012, China; hehongli@163.com

**Keywords:** risky play, teacher rating scale, reliability and validity, pre-schoolers, Anji play

## Abstract

To help teachers better evaluate the level of risky play for pre-schoolers, the present study aimed to develop a Teacher Rating Scale of Risky Play (TRSRP) for 3–6 years Chinese Pre-schoolers. The scale was administered to a pre-schooler sample consisting of 1376 children (*M*_age_ = 57.53 months, *SD* = 10.38; 54.30% boys; 44.80% only child) recruited from Anji play kindergartens in Hangzhou, China. The psychometric properties of the instrument were examined. The reliability of scale was reported by calculating internal consistency. The construct validity of the scale was investigated by exploratory factor analysis and confirmatory factor analysis. The final 7-item measure was structured into two subscales: play with great heights and play with high speed. The results suggested that the TRSRP has acceptable internal consistency and construct validity and can be used as an effective tool to measure the level of risky play for 3–6 years pre-schoolers in China’s Anji play kindergartens.

## 1. Introduction

Risky play refers to thrilling and exciting forms of physical play that involve uncertainty and a risk of physical injury [1]. Most pre-schoolers engage in risky plays in some way. During playtime, especially outdoor play, young children crave to look for risks and make risky decisions, so as to experience the rewards of positive emotions such as joy and excitement, which is actually a natural motivator for participation in risky play [2]. Children not only thought of risky activities as “adventurous”, “stimulating”, and “cool,” but also pointed out a variety of physical and emotional sensations caused by fear (e.g., having butterflies in their stomachs and feeling happy and scared at the same time) [3]. The emergence and development of Anji play in China has brought a new concept to the field of preschool education, and gradually changed the fact that China restricts children to engage in risky play.

### 1.1. Anji Play

Anji play is the abbreviation of the kindergarten game-curriculum mode with Chinese local characteristics carried out by Anji County in Huzhou City, Zhejiang Province, China. The Anji play education mode clearly and heavily emphasizes “play”. Before 2000, most kindergartens in China were in a state of “no play”. In 2001, the “Guidelines for Kindergarten Education (Trial)” clearly pointed out that kindergartens need to “take play as the basic activities” [4]. Thus, the status of play in kindergartens can be truly confirmed. However, the play at that time was a “fake play” consisting of formalism and utilitarianism. Anji play kindergarten recognizes the drawbacks of “fake play”, and encourages a revolution in play, namely “letting children play and discover children through play”, so as to look for an educational model of “true play” that can inspire children’s lives [5]. After 22 years of revolution in play, Anji play has consciously embarked on the road of “game-based curriculum” [6]. Anji play implements the views that “children are capable active learners”, “teachers are observers, listeners and interlocutors”, and, regarding education, “real play is equal to real learning” and “the curriculum arises naturally in children’s activities”. Under the guidance of such an educational philosophy, the venues for outdoor activity of Anji play kindergarten are challenging and natural, where pre-schoolers play for at least 90 min every day outdoors. Teachers have prepared buckets, wooden boxes (cubes), ladders, mats, longboards and other typical materials of Anji play for children so that they can boldly play their favourite risky play in outdoor activities. The five key words of Anji play are “love, risk-taking, joy, engagement and reflection”, which also fully demonstrates the effectiveness of the Anji play model on young children [5].

The education model of Anji play has developed rapidly and has received extensive attention in the field of preschool education. The “Exploration and Practice Based on Anji play Model” was awarded the “First Prize of National Teaching Achievement Award for Basic Education” in 2014, leading a wave of learning from Anji play across China. In 2018, the Zhejiang Provincial Department of Education established 103 Anji play kindergartens. In 2019, Hong Kong established the Anji play learning circle, and Macau set up two Anji play kindergartens. In 2020, the Zhejiang Provincial Department of Education established the second batch of Anji play kindergartens, totalling 117. Up to 2020, 14,000 children in Zhejiang were enrolled and Anji play had been spread to over 100 public schools in all the provinces of China. The key points of the work of the Ministry of Education of China in 2021 clearly stressed that the implementation of Anji play be promoted on a national scale [7]. Internationally, in 2013, Dr. Chelsea Bailey, PhD in Curriculum and Teaching from the University of Wisconsin, Madison, took the lead in drafting the “‘Anji play’ International Promotion Plan”, and started the overseas promotion of Anji play. In 2016, Madison, Wisconsin took the lead in implementing the Anji play course. In 2017, Anji play entered the Belt and Road countries. In 2019, there were more than 30 overseas practice institutions of Anji play courses. In 2020, the latest report “School of the Future: Defining a New Education Model for the Fourth Industrial Revolution”, released by the World Economic Forum, selected 16 promising educational models worldwide, and Anji play was one of them [8].

### 1.2. Risky Play among Pre-Schoolers

The possible benefits of risky play regarding children’s development have been of particular interest to researchers in the last few decades. Risky play has an important impact on the physical and mental health of young children. Some of this research indicates that risky play can improve pre-schoolers’ physical activity and motor competence [9,10], provide them with opportunities to learn new skills, enhance their ability to assess and manage risk appropriately [11], arouse their exciting and stimulating feelings [2], and meet their needs of adventure, thus helping them to cultivate the spirit and will to face difficulties in an active way [12]. However, young children who lack the experience of risky play will encounter difficulties to acquire the risk management skills necessary for growth [13]. In one study, young children in the experimental group who received a 14-week risk play intervention improved their risk detection and ability, raised self-esteem, and reduced conflict sensitivity compared to pre-intervention performance, as well as those in the control group [11]. At the same time, children’s muscle strength and coordination skills are enhanced through experiences such as climbing a tree or rolling down a mountain [10,14]. Risky play can also help children better perceive possible threats in their environment and help them make the necessary decisions about dangerous situations, which will better develop their resilience, independence and self-confidence in the long run [13,15]. When children engage in risky play, they tend to break boundaries, try new ways of doing things, be innovative in their thinking, and make decisions about their actions. Making such decisions helps improve children’s problem-solving skills and creativity, which are seen as highly necessary learning abilities for today’s learners [16]. Meanwhile, prior studies have also indicated that the overprotection of children may have negative effects on their development. For example, limiting the scope of risky play and pre-schoolers’ autonomy increases anxiety, both in childhood [17,18], and in adolescence and adulthood [19,20].

A child’s acceptance and attitude towards risk play, to a large degree, depend on culture. Studies carried out in Central Africa described some common parenting practices in this area, yet the risk levels of those practices, such as eight-month-old babies playing with knives and helping chop tinder for the household fire [21], or two-year-old babies roaming alone in villages and surrounding forests and fields [22], are unacceptable in current Western culture. However, comparative studies have shown that there are also differences between Western countries. New et al. compared ECEC teachers in Norway, Sweden, Denmark, Italy, and the United States and found that European teachers were not as worried about their children’s risk-taking behaviour as their American colleagues [23]. Similarly, Little et al. found that Scandinavians, particularly ECEC practitioners in Norway, were more casual towards children’s risky play than those in Australia [24]. Liu compared the risky play in Norway and China, and found that the risky play in Norwegian kindergartens were the most mature; the risky play in Anji play kindergarten obtained the initial results and children had plenty of time and opportunities to experience risky play. The teachers there also had a basic understanding of risky play under the guidance of the concept of kindergarten and gradually formed their own views in practice; Shanghai kindergartens gradually tried to let go of children’s risky play, but the opportunities for young children to participate in the risky play were limited [25]. This is often related to the cultural beliefs and values of countries, as well as to the different importance attached to outdoor play and learning between countries [23,26].

From the perspective of Hofstede’s Cultural Dimensions, China has a high level of uncertainty avoidance culture. This culture tends to rely very strongly on their set rules and ways of doing things. It may be seen as an attempt to control as much of the uncertainty as possible [27]. Thus, risky play in China is not encouraged. In addition, the current educational environment in China emphasizes the safety of students, thus putting students at risk of injury is regarded as a serious lack of care on the part of teachers. Thus, there are few kindergartens in China that provide opportunities for children to engage in risky play. Even when children start risky play spontaneously, teachers tend to be very nervous and immediately stop and warn children to stop. In this way, young children’s autonomy in risky play is limited [12].

### 1.3. Measurements of Risky Play

Kaarby found that climbing steep slopes and sliding down, jumping off top stones, keeping balance on fallen trees, climbing trees, keeping balance in high places, using a stick for fencing, etc. are common risky play for children [28]. Sandseter divided risky play into six categories through an observation of children’s outdoor games in Norwegian kindergartens and interviews with teachers, namely play with great heights, play with high speed, playing with dangerous tools, play near dangerous elements, rough-and-tumble play and play where children can “disappear”/get lost [29]. Kleppe et al. conducted an observational study on the risky play for children aged 1–3 years and believed that risky play should also include play with impact and vicarious play [30]. Little and Eager divided risk-taking behaviours into nine categories by observing children’s risky play in community parks, according to the difficulty of challenges and the correctness of behaviours for children, namely, refusing to take risks, trying to take risks, risk-free, no risk, low risk, medium risk, and high risk [31]. Among them, risk-taking behaviours from low to high level can be negative or positive. For example, the low-level risks were divided into active ones and negative ones. Yurt and Keleş provided young children with three levels of cartoons on risky play based on Sandseter’s study. The cartoons were themed sitting on a branch (play with great heights), swinging (play with high speed), playing near a fire (play near dangerous elements), using scissors (using dangerous tools), and the plays of each theme have three levels for children to choose to measure children’s preference for the level of risky play [32].

### 1.4. The Current Study

This study aimed to develop a Teacher Rating Scale of Risky Play (TRSRP) for 3–6 year pre-schoolers in the context of Chinese culture. Based on the literature review, the existing measurements for risky play, including the categories and subcategories of risky play [29], categories of risk-taking behaviour [31], and the children’s risk preferences/the level of risk preferred by children [32], are all based on western culture. However, China’s cultural context and educational environment are quite different from that of the West. Whether these risky play measurements established are applicable to China needs to be investigated.

We intended to develop the TRSRP in Anji play kindergartens of China for two reasons. In the current study, Anji play kindergartens refers to the kindergartens that apply the Anji play educational model and follow its typical education practices. First, Anji play encourages play, especially risky play; “risk-taking” is one of the five highlights of the educational mode of Anji play. Thus, Anji play kindergarten can offer the appropriate circumstances for risky play study in China. Second, the rapid development of Anji play requires corresponding measures for risky play to assist the practice of Anji play. The educational mode of Anji play is being promoted across the country and even the world, and the risky play of pre-schoolers has attracted much attention. However, teachers always have doubts about risky play in Anji play, for it has problems such as being unable to accurately recognize the threshold of children’s risk tolerance, and to measure the level of risky play for pre-schoolers of different ages, which affects teachers’ support for and guidance on risky play for pre-schoolers. Therefore, the value of risky play cannot be realized in the end. For teachers, measuring risky play effectively can help them to provide effective support and guidance for children engaging in risky play. 

Thus, the current study intended to develop a risky play measurement that can effectively measure the level of risky play for pre-schoolers, so as to provide support for teachers to further understand the level of risky play and guide risky play for pre-schoolers. Developing a measurement for risky play for pre-schoolers, according to the principles and procedures of psychometrics, can not only evaluate the level of risky play for of Anji play kindergartens in China, but also provide necessary reference for the evaluation of risky play for pre-schoolers in other kinds of kindergartens in China and countries with a similar culture context.

## 2. Materials and Methods

### 2.1. Procedures of the TRSRP Construction

The TRSRP was constructed in three steps. The first step included the development of a type and subtype of risky play. The second involved the development of level descriptions of each subtype of risky play, Finally, the third step included the evaluation and revision of the TRSRP. In addition to the literature review, observations and interviews in three kindergartens were carried out in this process, so as to offer a reference for the construction process.

1. Observations: Through non-participatory and participatory ways, the outdoor activities of pre-schoolers of 9 classes in three Anji play kindergartens were observed for 6 weeks. In China, there are typically three levels of classes: Lower Kindergarten Class for Aged 3–4 children (Xiaoban), Middle Kindergarten Class for Aged 4–5 children (Zhongban), and Upper Kindergarten Class for Aged 5–6 children (Daban). In each kindergarten, three classes (one Xiaoban, one Zhongban, one Daban) participated in this stage. The teachers in each class were instructed to record video clips of children’s risk play (the behaviour they regarded as risk in the play). Relevant pictures were taken to indicate the details of the setting and observations were recorded in written form. In sum, each set of risky play behaviour record included video clips, 1–2 pictures and an observation record. The teachers of each class collected about 22 sets of records. A total of 200 sets of risky play were collected. Those records were used for the classification and level description construction.
2. Interviews: The principle and five teachers in each kindergarten, covering three levels of classes (Xiaoban, Zhongban, Daban), were invited to participate in the interview. A total of 15 teachers and 3 principals of three Anji play kindergarten were interviewed. The content of the interviews mainly concerned the typical performance of children in play of bucket/vertical jump/balance beam walking/climbing/slide/high-speed swing/high-speed ride/high-speed running. Those interviews were used for the construction and modification of the TRSRP.

#### 2.1.1. Type Determination

The widely recognized classification scale of risky play developed by Sandseter divided risky play into six categories, including (a) play with great heights, (b) play with high speed, (c) play with dangerous tools, (d) play near dangerous elements, (e) rough-and-tumble play, and (f) play where children can “disappear”/get lost [29]. At the same time, Sandseter also emphasized that play with great heights, play with high speed, and rough-and-tumble play are the most common risky play in kindergarten [33]. Based on the classification, this research will use methods of observation and interview to understand the actual situation of Anji play kindergarten in China and make appropriate modifications to the types of risky play.

First, all kindergartens in China are equipped with walls or fences, so there is no situation where children are allowed to go out alone, and children get lost. In the outdoor play for pre-schoolers in kindergarten, the children sometimes get into the drum, the wooden box, or put the mat upright and hide themselves in the mat. At this time, the children seem to be out of the teachers’ sight for a short time. In fact, materials such as buckets and wooden boxes are not completely enclosed. Teachers can still see the activity play of children in the buckets and wooden boxes, where children will not stay for a long time. Therefore, the play does not get to the point of risk. For the above reasons, risky play in this study does not include play where the children can “disappear”/get lost. 

Second, Chinese kindergartens do not allow young children to play near dangerous elements—where children can fall into or from something, such as cliffs/deep water/burning fire pits, etc., so risky play in this study do not include play near dangerous elements.

Third, in the outdoor play of Anji play kindergarten, teachers hardly provide dangerous tools such as knives, hammers and saws for children to use. A prior investigation on risky play in Anji play kindergarten found that there is no play with harmful tools and play near dangerous elements [25].

Fourth, teachers almost unanimously believe that rough-and-tumble play should be stopped as soon as it is discovered. It is pointed out that children at a young age have not yet mastered their own strength, so they can easily make aggressive play and thus hurt their peers. In addition, even if young children interact with their peers through body language to invite them to play together, in many cases their peers do not realize that this is a signal of an invitation to play, and they often think that the other party is hitting them on purpose. Due to strict control from teachers, rough-and-tumble play is extremely rare in Chinese kindergartens.

Based on the above analysis, this study finally constructed risky play as two types: play with great heights and play with high speed.

#### 2.1.2. Development of Risky Play Sub-Types and Level Descriptions

After observing children’s outdoor play and interviewing teachers, this study obtained possible subtypes under play with heights and play with high speed. Then, 20 teachers from Anji Play kindergartens were invited to evaluate the popularity of each subtype of play based on daily observations. The findings suggest that 100% of teachers think bucket is a very common play, 85% think vertical jump, balance beam walking and climbing are very common plays and 75% think high-speed swing, high-speed slide, high-speed ride and high-speed run are very common plays. In this way, the structure framework of the risky play scale was constructed (Table 1).

Level descriptions for each sub-types of risky play were developed by combining the data from observations and interviews. The non-participatory observation mainly observed how children engage in risky play in daily life, and what level of risky play children of each age group generally participate. In order to examine the level of risky play for pre-schoolers in different age groups in more detail, the current study also set the scenes of daily risky play, engage in the risky play with pre-schoolers as good friends and invite them to show the highest level of such play. After understanding the different performances of pre-schoolers in different types of risky play, level descriptions for each sub-types of risky play were summarized, and the Teacher Rating Scale of Risky Play for pre-schoolers aged 3–6 was developed.

#### 2.1.3. Evaluation of Content Validity and Finalization of TRSRP

To ensure the content validity of TRSRP, a total of eight experts (two preschool education experts, one sports health education expert, and five front-line educators) were invited to evaluate the scale. The evaluation consisted of two sections. First, the evaluation of the structure collected an expert evaluation of the structure of the scale, including the types and subtypes of risky play. Second, the rationality of the level descriptions was evaluated. Experts rated on a 5-point Likert-type scale ranging from 1 (very unreasonable) to 5 (very reasonable). The results indicate that the structure of the scale was good, with all evaluation scores above 4.13. For the level description (see Appendix A), the five levels of descriptions were numbered and labelled (1 = Elementary to 5 = Advanced). The evaluation score for three level descriptions were relatively low: description for Level 3 Intermediate (score: 3.88) and Level 5 Advanced (score: 3.63) of high-speed swing and Level 4 Upper-intermediate of high-speed ride (score: 3.88). However, the evaluation score for other subtypes were higher than 4.13, indicating a good enough level. Feedbacks regarding low evaluation scores were collected and the revised recommendation was summarized as follows. First, for safety, the vertical jump at any height needs a protective mat put on the ground. Second, the balance beam at any height also requires a protective mat on the ground for safety. It is believed that although some children have the ability to quickly walk over a balance beam of a certain height, there are situations where other children accidentally bump into the balance beam during outdoor free play, which may cause serious injury. Third, the maximum amplitude of the swing game needs to be changed from 90 degrees to 75 degrees. For safety reasons, the swing customized by the kindergarten cannot be swung to 90 degrees.

We modified the scale in response to the recommendations from experts, and the Teacher Rating Scale of Risky Play for 3–6 years Pre-schoolers was completed. It is a teacher-rating scale for measuring risky play for young children aged 3 to 6. The scale contains two factors: play with great heights (4 items) and play with high speed (4 items). Play with great heights includes bucket, vertical jump, balance beam walking and climbing, and play with high speed includes high-speed swing, high-speed slide, high-speed ride, and high-speed running. Each item is divided into five levels with specific level description. The scores of the two separate factors (average score of its corresponding subtypes) and the total score (average score of two factors) ranged from 1 to 5, with the higher score indicating higher risky play level.

### 2.2. Participants

This study collected data from four Anji play kindergartens in Hangzhou City, Zhejiang Province, China. A total of 1376 pieces of valid data were obtained by cluster sampling. They were divided into two samples.

Sample 1 (*N*_1_ = 220, *M*_age_ = 57.66, *SD* = 10.30; 50.5% boys; 60.0% is only child who is the only child of the family) included 71 children from Xiaoban (*M*_age_ = 45.38 months, *SD* = 3.56; 33.8% boys; 67.6% only child), 74 from Zhongban (*M*_age_ = 57.84 months, *SD* = 3.62; 55.4% boys; 55.4% only child), 75 from Daban (*M*_age_ = 69.09 months, *SD* = 3.46; 61.3% boys; 57.3% only child).

Sample 2 (*N*_2_ = 1156, *M*_age_ = 57.50 months, *SD* = 10.40; 55.0% boys; 41.9% only child), of which 381 are from Xiaoban (*M*_age_ = 45.83 months, *SD* = 4.02; 52.5% boys; 43% is only child), 400 from Zhongban (*M*_age_ = 57.97, *SD* = 4.43; 55.3% boys; 45.3% only child), 375 from Daban (*M*_age_ = 68.85 months, *SD* = 5.34; 57.3% boys; 37.1% only child). 

### 2.3. Procedure

The study was reviewed and approved by the Ethics Committee of the first author. First, the study sent an invitation letter with necessary information about the study to the principal of the kindergarten. After approval, the kindergarten sent a notification to teachers inviting them to participate in the study. All participants signed the written consent form and acknowledged that their participation is voluntary and anonymous, and they can terminate the study at any time. Teachers were asked to evaluate the level of risky play for each young child in the class according to the requirements of TRSRP.

### 2.4. Statistical Analysis

All data analyses were performed using IBM SPSS Statistics 22 (IBM Corp., Armonk, NY, USA). First, item analysis and exploratory factor analysis were performed on samples 1. Items were analysed using the item-total correlation and the critical ratio. For EFA, we performed a principal component analysis using Varimax rotation. Second, CFA was performed on sample 2. For CFA, we assessed how well the model fits the data by chi-square statistics, comparative fit index (CFI > 0.90), Tucker–Lewis index (TLI > 0.90), standardized root-mean-square-residual (SRMR < 0.08) and root mean square error of approximation (RMSEA < 0.06) [34]. Thirdly, in order to test the reliability of the rating scale, this study used Cronbach coefficient for sample 2 to analyse the reliability of the internal consistency of the rating scale. When the Cronbach coefficient is greater than 0.70, the reliability of the rating scale is good [35].

## 3. Results

Before testing the validity and reliability of the TRSRP, item analysis was performed. All eight items met the criteria of psychometrics (*r* = 0.722~0.901, *p* < 0.01). In addition, the critical ratio was used to analyse the degree of differentiation of each item in the rating scale. All study subjects were ranked from highest to lowest overall scores. Then, those who scored in the top 27% were assigned to the high score group (*n* = 65), and those who scored in the bottom 27% were assigned to the low score group (*n* = 65). The independent sample *t*-test was used to compare the differences between the two groups of subjects on each item. The results showed that all 8 items met the criteria of psychometrics (*CR* = 11.29~25.94, *p* < 0.001). Therefore, we retained all 8 items for exploratory factor analysis.

### 3.1. Validity of the TRSRP

The validity of a test refers to the extent to which the test correctly measures the construct (or constructs) that it is supposed to measure [36]. The content validity and construct validity were examined.

#### 3.1.1. Content Validity

The basic procedure for evaluating content validity, as Murphy and Davidshofer suggest, consists of three steps: (1) describe the content domain, (2) determine the areas of the content domain that are measured by each of the test items and (3) compare the structure of the test with the structure of the content domain [37]. The procedures were reported in the item construction section above.

#### 3.1.2. Construct Validity

Exploratory Factor Analysis (EFA). The KMO value for the 8-item rating scale was 0.88. The Bartlett sphericity test showed that the correlation matrix was suitable for factor analysis (*χ*^2^ = 1668.53, *df* = 28, *p* < 0.001). EFA produced two significant factors with eigenvalues greater than 1. Item 4 (climbing) was deleted due to the low factor loadings on both factors (0.61 and 0.39, respectively). The remaining seven factor loadings ranged from 0.75 to 0.89, explaining 80.52% of the total variance. The results are shown in Table 2: the first factor contained four items, while the second factor contained another three items. The eigenvalues were 8.66 and 1.41, and the proportions of variance explained were 69.26% and 11.26%, respectively. There was a significant correlation between the two factors (*r* = 0.70, *p* < 0.01). These two factors are called “play with high speed “ and “play with great heights”.

**Figure 1:**
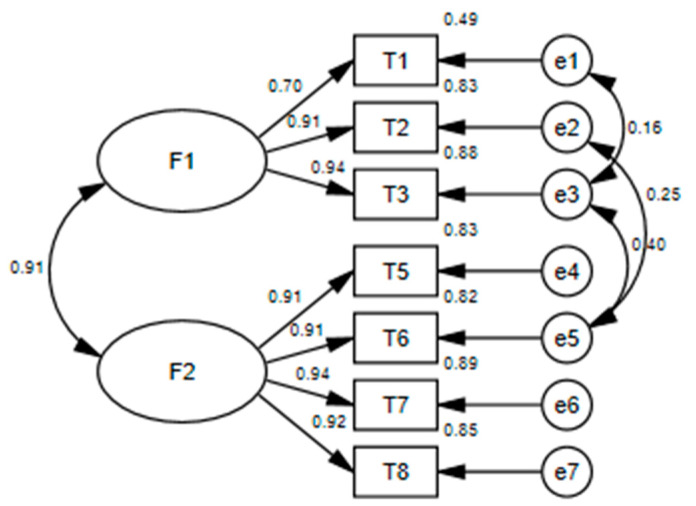
Path diagram for the CFA of the TRSRP. F1 = play with great height; F2 = play with high speed.

Confirmative Factor Analysis (CFA). To validate the factor structure determined by EFA, CFA was performed. We added the error covariances between item 1 and item 3, item 2 and item 6, and item 3 and item 6, as suggested by the modified indices. After adding the error covariance, the results showed that all fit indices meet the statistical criteria (see Table 3 and Figure 1), indicating the structure of the rating scale with two factors and 7 items (see Appendix A) has good fit indices and appropriate construct validity.

### 3.2. Reliability of the TRSRP

To test the reliability of the rating scale of risky play, an internal consistency test was performed. Internal consistency reliability refers to the fact that all items on the same scale consistently or reliably measure the same dimension [37]. The Cronbach’s coefficient for the entire rating scale was 0.95, and the Cronbach’s coefficients for factor 1 (play with great height) and factor 2 (play with high speed) were 0.89 and 0.96, respectively. Results indicated that the TRSRP has satisfactory reliability.

## 4. Discussion

The study developed a seven-item measurement of risky play for 3–6 pre-schoolers that is easily used by teachers to evaluate risky play levels for Chinese kindergarten pre-schoolers. The results of investigating preliminary internal consistency, construct validity data for an Anji play kindergarten pre-schooler sample supported the TRSRP as a reliable and valid measurement tool to measure the level of risky play for pre-schoolers.

The construct validity of this scale was analysed using EFA and CFA techniques. The EFA and CFA results for the TRSRP items provided evidence that the instrument has two factors which seem to fit the two-factor model designed above. Risky play is divided into play with great heights and high-speed play. Among them, the play with great heights refers to the plays played by children at a higher place, and there is a risk of falling from a height. Play with high speed refers to plays played at higher speeds by young children, with a risk of colliding with something or someone. The internal consistency of the TRSRP was 0.95 and the internal coefficients for those two subscales was 0.89 and 0.96, respectively. The results suggest that the TRSRP has acceptable internal consistency and construct validity. It can be used to help Kindergarten teachers to evaluate risky play for 3–6 pre-schoolers in China.

The TRSRP laid an important foundation for studies and teaching practices of risky play in China pre-schoolers. This study explores the structure of risky play for pre-schoolers in the context of Chinese culture. First, this two-factor structure funded in the current study was consistent with previous research findings that young children prefer play that allows them to experience a sense of great heights and high speed [31]. Risky play for pre-schoolers mainly involves play with high speed and play with great heights, and the former is the most common in risky play for pre-schoolers [12]. Second, the two factors identified in the current study was based on localization. The structure of the rating scale was based on the actual situation of Chinese kindergartens, and the level descriptions of risky play in this scale is based on a sample of children in China’s Anji play kindergarten. At the same time, the development of this scale can also provide a certain reference for measuring the level of risky play for pre-schoolers in other kindergartens in China and countries with similar culture background.

To our knowledge, this was the first study to develop a measurement of risky play for pre-schoolers, strictly in accordance with the norms and principles of psychometrics in the Chinese context. Three educational implications of the current study should be acknowledged. First, The TRSRP can effectively measure the level of risky play for pre-schoolers in China’s Anji play kindergarten, help teachers to more accurately understand the level of risky play for pre-schoolers in the class, provide effective support for children of lower-level risky play and discover the potential of children of advanced-level risky play. Second, the development of this rating scale can provide a certain reference for other kindergartens in China and kindergartens to evaluate the level of risky play for pre-schooler. Researchers can adapt the rating scale based on this study to make it suitable for preschool teachers in different countries and regions. Third, the development of this scale will help to deepen our understanding of risky play for pre-schoolers and promote the deepening of theory of children’s risky play. For example, future research can continue to explore the status of children’s risky play, the influencing factors and development stage of the level of children’s risky play, and the different developmental values brought by risky play at different levels to children on the basis of the current study.

However, this study still has some limitations. First, for the convenience of sampling, all the data in this study were collected in the Anji play kindergarten in Hangzhou, Zhejiang Province, China. Further research is needed to confirm the applicability of this tool in non-Anji Play kindergartens. As risky play is restricted in these environments, we may encounter “floor effect” in its application, which may result in low discrimination. Second, the concurrent validity index of the tool has not been tested, and it can be tested in the future. Third, the norms of a measurement are important for its usefulness in practice [36] and thus future research on external validity should be investigated in more comprehensive settings to set norms of TRSRP, especially the norms for different ages and genders. Finally, future research could also explore the development of a parental report scale of risky play for pre-schoolers to enable assessments from and comparison between multiple resources of a pre-schooler’s risky play.

## 5. Conclusions

Despite its limitations, the results of this study indicated that the Teacher Rating Scale of Risky Play has good reliability and validity and is a valid tool to assess the level of risky play for 3–6 years pre-schoolers in Anji play kindergartens in China. It enriches the cross-cultural research of the rating scale of risky play and provides support for teachers to evaluate risky play levels of pre-schoolers and to guide risky play.

## Figures and Tables

**Table 1:** The structure framework of risky play scale.

Types	Subtypes and Definitions
Play with great heights	Playing with buckets: a play that uses buckets as materials. Usually, pre-schoolers can push the buckets, crawl inside the buckets and walk on the buckets.
	Vertical jump: a play in which children stand on a platform of a certain height and jump down.
	Balance beam walking: a play in which children walk on balance beams of different heights.
	Climbing: a play in which climbing, and scrambling are the main movements.
Play with high speed	High-speed swing: a play in which children swing from low to high on a swing in a variety of positions.
	High-speed sliding: a play in which children use long boards to construct different slopes for sliding.
	High-speed ride: a play in which children use tricycles to ride at high speed.
	High-speed running: a play in which running is performed at a high speed.

**Table 2:** Standardized factor loadings of exploratory factor analysis of the TRSRP.

Factors	1	2
Factor 1		
Playing with buckets	0.82	
Vertical jump	0.89	
Balance beam walking	0.75	
Factor 2		
High-speed swing		0.87
High-speed slide		0.86
High-speed ride		0.89
High-speed running		0.84
Eigenvalues	1.41	8.66
% of the variance explained	11.26	69.26

**Table 3:** Model fit index of the TRSRP.

Model	*χ* ^2^	*df*	RMSEA	NFI	RFI	CFI	IFI	SRMR
Two-factor model	50.21	10	0.05	0.99	0.98	0.99	0.99	0.01

## Data Availability

The data presented in this study are available on request from the corresponding author.

## Appendix A

Instruction: The types of risky play and the 5 levels of each type are listed below, please select the appropriate level for the child.

**Table A1:** Teacher Rating Scale of Risky Play for 3–6 years Pre-schoolers.

Type and Level Descriptions
**Playing with buckets**
1. Elementary: pushing the buckets with hands, pushing the bucket with a partner, climbing in a single bucket, curling up and rolling in the bucket, playing the game of whack-a-mole with the bucket upright, etc.
2. Pre-intermediate: climbing in multiple buckets that have been arranged and combined, walking on multiple buckets that have been fixed and combined, etc.
3. Intermediate: with the protective mat in front or with the help of teachers or partners, try to stand on the bucket in various ways and start learning how to walk on the bucket.
4. Upper-intermediate: able to walk on buckets independently, flexibly and confidently.
5. Advanced: if there is a huddled companion in the bucket, the child can also walk on the bucket; able to jump to another bucket to continue walking; walk on the bucket hand in hand with the partner; while standing on the bucket, play other games, such as passing the ball, playing hula hoop and so on.
**Vertical jump**
1. Elementary: children observe the behaviour of their peers jumping from a height. They try to stand at a height of 40–50 cm and want to jump down, but they are always hesitant, and finally fail to jump down.
2. Pre-intermediate: a protective mat is put on the ground, and the child can jump down from a height of 70–80 cm.
3. Intermediate: a protective mat is put on the ground, and the child can jump down from a height of 100–110 cm.
4. Upper-intermediate: a protective mat is put on the ground, and the children can jump down from a height of about 130–140 cm, flexibly and confidently.
5. Advanced: a protective mat is put on the ground, and the children can jump down from a height of about 150 cm or even higher.
**Balance beam walking (The balance beam is 200 cm long, and 20 cm wide)**
1. Elementary: with a protective mat on the ground, the child tries to walk on a balance beam at a height of 40–50 cm from the ground, with or without the support of a companion or an adult.
2. Pre-intermediate: when there is a protective pad on the ground, the child can walk alone from the balance beam at a height of 60 cm to 70 cm above the ground, but he or she may walk slowly and is slightly nervous.
3. Intermediate: when there is a protective pad on the ground, the child can walk independently, flexibly and confidently from a balance beam with a height of 80 cm to 90 cm.
4. Upper-intermediate: when there is a protective pad on the ground, the child can challenge to walk on a balance beam with a height of 100–110 cm, but he or she may bend his or her waist to walk slowly because of fear.
5. Advanced: when there is a protective pad on the ground, able to walk through the balance beam of 120–130 cm above the ground independently, flexibly, confidently and quickly.
**High-speed swing (Take swing as an example)**
1. Elementary: children sit on a swing with the help of their peers or teachers, and the swing range is 30–45 degrees.
2. Pre-intermediate: children sit on a swing with the support of their peers or teachers, and the swing range is 45–60 degrees.
3. Intermediate: children sit on a swing with the support of their peers or teachers to swing, and the swing range is 60–75 degrees.
4. Upper-intermediate: the ways of swinging are more diversified. For example, with the support of peers or teachers, one child can squat on the swing, and the swing range is about 45 degrees; or stand on the swing, and the swing range is about 30 degrees; or a fast circular swing; or a contest of who can swing fast and high.
5. Advanced: there is a protective cushion in front, and the child flies out of the swing and jumps on the cushion, and even make a competition of “see who can fly farther” or perform aerial modelling to see whose pose is more difficult.
**High-speed slide (Take slide as an example. he long board is 200 cm long and 20 cm wide.)**
1. Elementary: slide down from a self-built slide with a height of 50–60 cm.
2. Pre-intermediate: slide down from a self-built slide with a height of 70–80 cm.
3. Intermediate: slide down from a self-built slide with a height of about 90–100 cm.
4. Upper-intermediate: slide down from a self-built slide with a height of 110–120 cm.
5. Advanced: slide down a self-built slide of 150 cm or higher.
High-speed ride (Take tricycle play as an example)
1. Elementary: children can ride at a moderate speed (1 m/s speed) but cannot turn flexibly and avoid obstacles.
2. Pre-intermediate: young children can ride at a relatively fast speed (1.5 m/s) on complex road (such as steep slopes, road with obstacles or narrow road), but need to slow down for safe passage when meeting obstacles or narrow sections.
3. Intermediate: able to compete with peers to see who can ride the fastest; or play a chase game by bike; or while riding a bike, a partner is pushing a tricycle behind to maximize the riding speed (greater than 1.5 m/s).
4. Upper-intermediate: it is possible to easily ride on complex sections of road (such as steep slopes, sections with obstacles or narrow sections) at a speed of more than 1.5 m/s with people or loads. Even if encounter obstacles or narrow sections, the child can also maintain the original speed and control the handlebars for safe riding.
5. Advanced: after the front wheels of several tricycles are stuck in the carriage of the preceding tricycle in a row, each child sits on his own tricycle, and there may be children behind him who are pushing the tricycle, trying to reach the fastest speed to start the “train”.
**High-speed running**
1. Elementary: run at a speed of 2.5 m/s, mainly in small steps with uneven stride, no rhythm, heavy footsteps, and heels on the ground. Two arms cannot naturally swing with the movements of the feet, and the ability to run fast is obviously insufficient.
2. Pre-intermediate: run at a speed of 3 m/s, with heels on the ground, and small stride, and the upper and lower limbs can cooperate more harmoniously, with a certain ability to run fast;
3. Intermediate: run at a speed of 3.5 m/s, the arms and lower limbs are out of position, the elbow joints and the legs are almost fully extended, and the ability to run fast and dodge is improved.
4. Upper-intermediate: able to run at a speed of 4 m/s, the forefoot is on the ground, the arms and lower limbs are out of position, the arms swing obviously, the stride is large and rhythmic, the movements are more coordinated, the flight phase is more obvious, there is a significant improvement in the ability to control the running direction and run, and it is more flexible to turn around, stop and dodge during high-speed running.
5. Advanced: when carrying certain items with both hands, such as a basketball, the child can still run left and right at a speed of 4 m/s, and it is more flexible to turn around, stop and dodge during high-speed running.
